# A novel pre-clinical antibacterial pipeline database

**DOI:** 10.1371/journal.pone.0236604

**Published:** 2020-07-28

**Authors:** Sarah Paulin, Richard A. Alm, Peter Beyer

**Affiliations:** 1 Department of Global Coordination and Partnership, Antimicrobial Resistance Division, World Health Organization, Geneva, Switzerland; 2 CARB-X, Boston, MA, United States of America; Nanyang Technological University, SINGAPORE

## Abstract

The clinical pipeline continues to be insufficient to contain antimicrobial resistance, and further investment and research is needed to ensure that a robust pipeline is built to treat the WHO priority pathogens list of antibiotic-resistant bacteria. To shed light further upstream on the preclinical pipeline the WHO has undertaken a review of the antibacterial preclinical pipeline and published the data of all identified projects in a publicly accessible database. The database captures 252 unique antibacterial agents in preclinical development being developed by 145 individual institutions, of which the majority are smaller biotech companies and academic institutions. There is a higher degree of innovation in the preclinical pipeline with a significant number of non-traditional approaches being pursued. For even a fraction of these projects to reach clinical development or the market, there is a need to shift the market dynamics for new antibacterials through the identification of new solutions beyond push and pull incentives.

## Introduction

Antibiotic resistance continues to be a global public health issue causing a heavy burden on health-care systems. In Europe alone, it is reported that antibiotic-resistant infections result in approximately 33,000 deaths annually [1], and 35,000 deaths annually in the USA [2]. Although the emergence of resistance is a natural phenomenon in bacterial pathogens, this has been accelerated through overuse and misuse of existing antibiotics resulting in treatment failures for both common and complicated infections. In order to identify the priority resistant bacteria for which new treatments were urgently needed, in 2017 the World Health Organization (WHO) published the WHO priority pathogens list to focus research and development efforts on existing and future treatment gaps [3]. As identified in the 2019 update of the WHO clinical antibacterial pipeline analysis, the current clinical antibacterial pipeline remains insufficient to counter the rise and spread of resistant bacteria [4]. It is dominated by new agents of existing antibiotic classes offering relatively incremental improvement with very few new innovative products being developed against the critical priority pathogens. In contrast to the clinical pipeline it is much more difficult to get a comprehensive overview and assess the composition and societal value of the preclinical pipeline. To provide more transparency, WHO has undertaken a broad review of the antibacterial preclinical development projects and published the data of all identified projects in a WHO publicly accessible database that complements an earlier review by Theuretzbacher et. al. that was based on anonymized data [5]. The data is available on the WHO Global Observatory for Health Research and Development and will be updated on an annual basis [6].

## Materials and methods

### Overview

The methodology for the WHO preclinical pipeline data call and subsequent establishment of the online preclinical database was developed through consultation with various experts in the R&D field including antibacterial R&D funders and small and medium size enterprise (SME) and associations of pharmaceutical companies. An online data call was organised, the submitted data cleaned, analysed and published on the WHO Global Observatory for Health Research and Development.

### Ethics statement

The research did not involve human participants and thus did not require ethical approval.

### Scope

In scope for the WHO preclinical pipeline data call were antibacterial projects that target any of the bacteria on the WHO priority pathogens list, *Mycobacterium tuberculosis* or *Clostridioides difficile*. All projects that are in lead optimization, preclinical candidate selection, or investigational new drug application (IND)/ clinical trial authorization (CTA) enabling studies were included. The inclusion criteria covered projects in the following categories: direct-acting traditional small molecules, repurposed non-antibiotics and antibiotics from animal to human use, and novel combinations, antimicrobial peptides, anti-virulence agents, biologics, decolonization agents, immunomodulators, microbiome-modifying agents, phage and phage-derived products, and potentiators and enablers. Excluded from the scope were vaccines, diagnostics, antifungals, antivirals or anti-parasitics, wound care agents, nonspecific supportive treatments, medical devices, and industrial and animal use agents.

### Data collection

An online data call was developed to collect non-confidential project level data on the preclinical pipeline projects which was supplemented by a desktop review. The online data call was developed based on the WHO DataCol platform which also allows for the electronic uploading of data including PDF documents for supporting information along with specific data entries. The DataCol had the following mandatory input fields: institution, name of responder, email address, product name or descriptor, preclinical phase (drop down options), product type (multiple choices possible) and the following voluntary input fields: spectrum (drop down options), indication, mode of action, route of administration, and infection stage targeted. The data call was open from 18 January to 18 April 2019 on the WHO website. Specific reviews of the preclinical pipeline landscape in China, Japan and Russia were also undertaken along with request for information from R&D funders and stakeholders (Access to Medicine Foundation, BEAM Alliance, BIO, CARB-X, REPAIR Impact Fund, Global Antibiotic Research and Development Partnership (GARDP), Global AMR R&D Hub, IFPMA, JPIAMR, Needham & Company, NIH/NIAID, TB Union, Pew Charitable Trusts, REPAIR Impact Fund, TB Alliance, Treatment Action Group) and supplemented with publicly available information along with a desktop review. The search terms used for the desktop review were preclinical OR lead optimization OR preclinical candidate combined with ‘antibacterial’ as well as review of public websites for companies that submitted information.

## Results

Following the cleaning of the data collected including removal of duplicates and verification of data obtained, 252 unique antibacterial agents in preclinical development developed by 145 individual institutions. The dataset was made publicly available on the WHO Global Observatory for Health R&D and will be updated on an annual basis [6].

### Institutions

**Fig 1:**
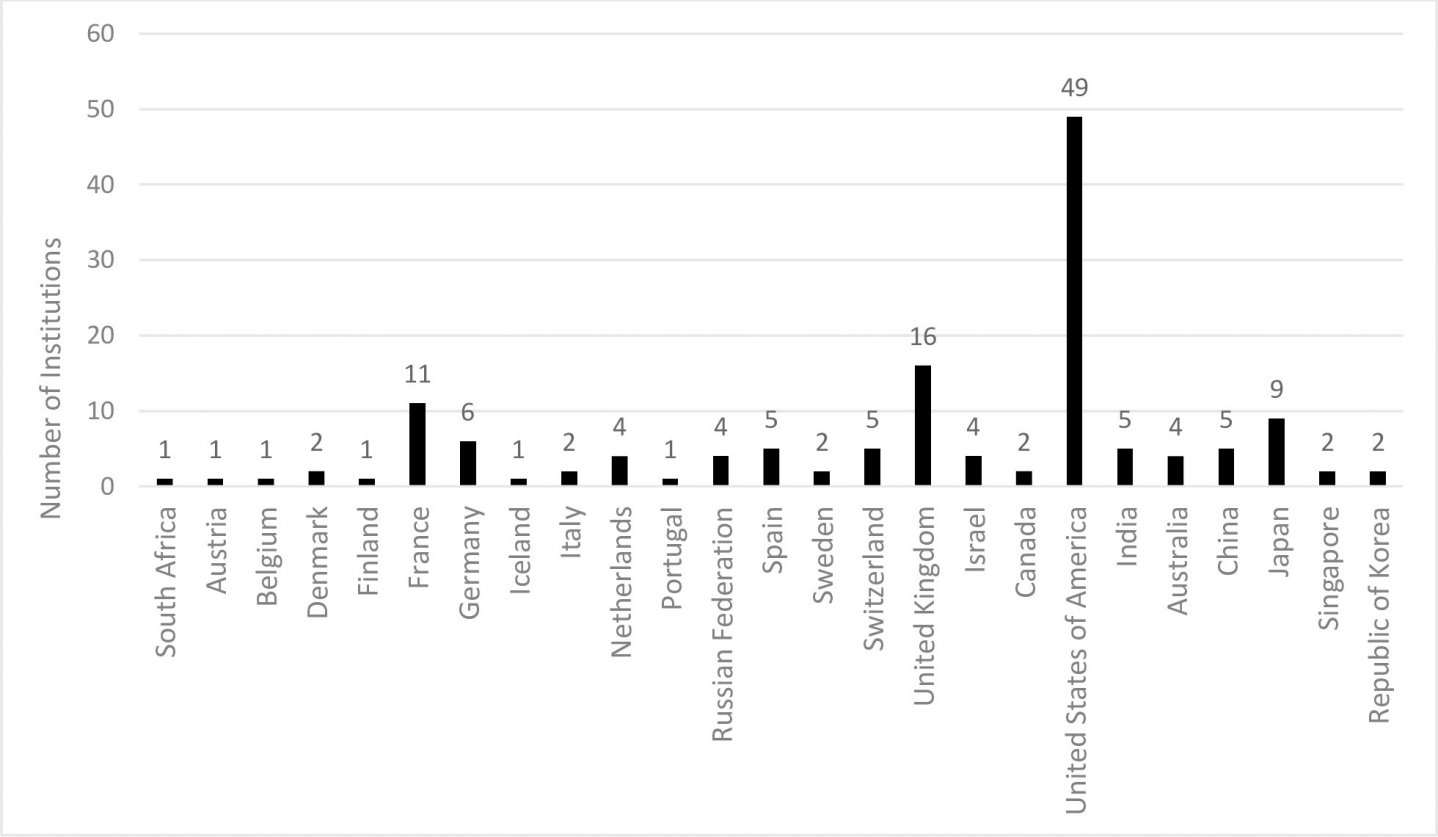
Geographical distribution of the institutions with active preclinical pipeline projects.

The 145 institutions that were included in the WHO database have a wide geographical variation with 66 (45.5%) of the institutions in the WHO European Region, 51 (35.2%) in the Region of the Americas, 22 (15.2%) in the Western Pacific Region, 5 (3.4%) in the South-East Asia Region and 1 (0.7%) in the African Region (Fig 1). The USA had the most institutions (n = 49, 34%) with preclinical pipeline projects for a single country. The majority of the institutions were classified as commercial companies (78.6), followed by academic institutions (18.6%) and foundations (2.8%). In addition the majority of the commercial companies are SMEs (n = 106/114, 93%) of which 91% (n = 96/106) were small enterprises (<50 employees).

### Preclinical projects

**Fig 2:**
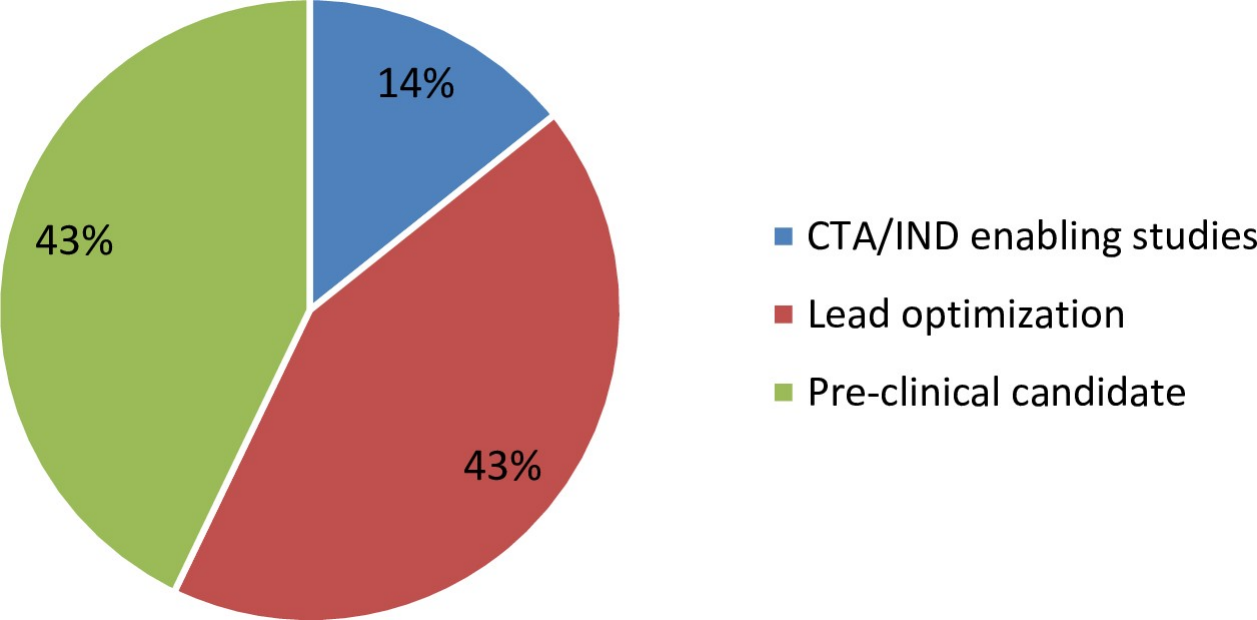
Percentage of distribution of pre-clinical pipeline projects across the pre-clinical development stages; lead optimization, pre-clinical candidate selection, CTA/IND-enabling studies.

The 252 preclinical pipeline projects that were identified and made available in the WHO database were aggregated based on the their self-reported pre-clinical pipeline stage, with 43% in lead optimization, 43% in pre-clinical candidate selection and 14% in CTA/IND-enabling studies (Fig 2).

The projects were also classified into five broad modality categories of which repurposed agents and non-traditional approaches were then further subcategorized as shown in Table 1.

**Table 1:** Categorization of preclinical pipeline projects.

Categories	Total (%)
**Direct-acting small molecules**	**108 (42.9)**
**Antimicrobial peptides**	**27 (10.7)**
**Novel combinations**	**9 (3.6)**
**Repurposed agents**	**18 (7.1)**
• Animal to human	2
• New application	11
• Non-antibiotics	5
**Non-traditional approaches**	**90 (35.7)**
• Anti-virulence agents (small and large molecules)	18
• Biologics	9
• Decolonization agents	3
• Immunomodulators	11
• Microbiome-modifying agents	1
• Phage/phage-derived products	28
• Potentiators/enablers (resistance-modulating, penetration-enabling)	17
• Other	3
**Total**	**252 (100)**

The number of non-traditional approaches being pursued was significant. There was a total of 108 that were traditional direct-acting small molecules, but closely followed in number by 90 non-traditional approaches. The largest sub-groupings of the non-traditional approaches were phage and phage derived products (n = 28), followed by 18 anti-virulence agents and 17 potentiators and enablers. Furthermore, of the 108 direct-acting small molecules the majority (n = 51) were in lead optimization with only 13 projects in CTA/IND-enabling studies. It is the similar trend in the non-traditional approaches where 36% (n = 40) are in lead optimization, 35% (n = 39) in preclinical candidate selection phase and only 10% (n = 11) in CTA/IND-enabling studies. These distribution of projects in these self-reported project stages likely indicate either the attrition rate of antibacterial projects [7], which is inherently high, or representative of the number of newer programs entering the pipeline.

The study also reviewed mode of action and the indication of the 252 preclinical pipeline projects. In discovery and preclinical phases, depending on the starting point of the program, the mode-of-action may not be fully elucidated or may still be regarded as proprietary. 32% of the 252 pre-clinical pipeline projects have “other” or “not disclosed” mode of action, followed by 20% cell wall synthesis and 14% cell membrane targeting.

**Fig 3:**
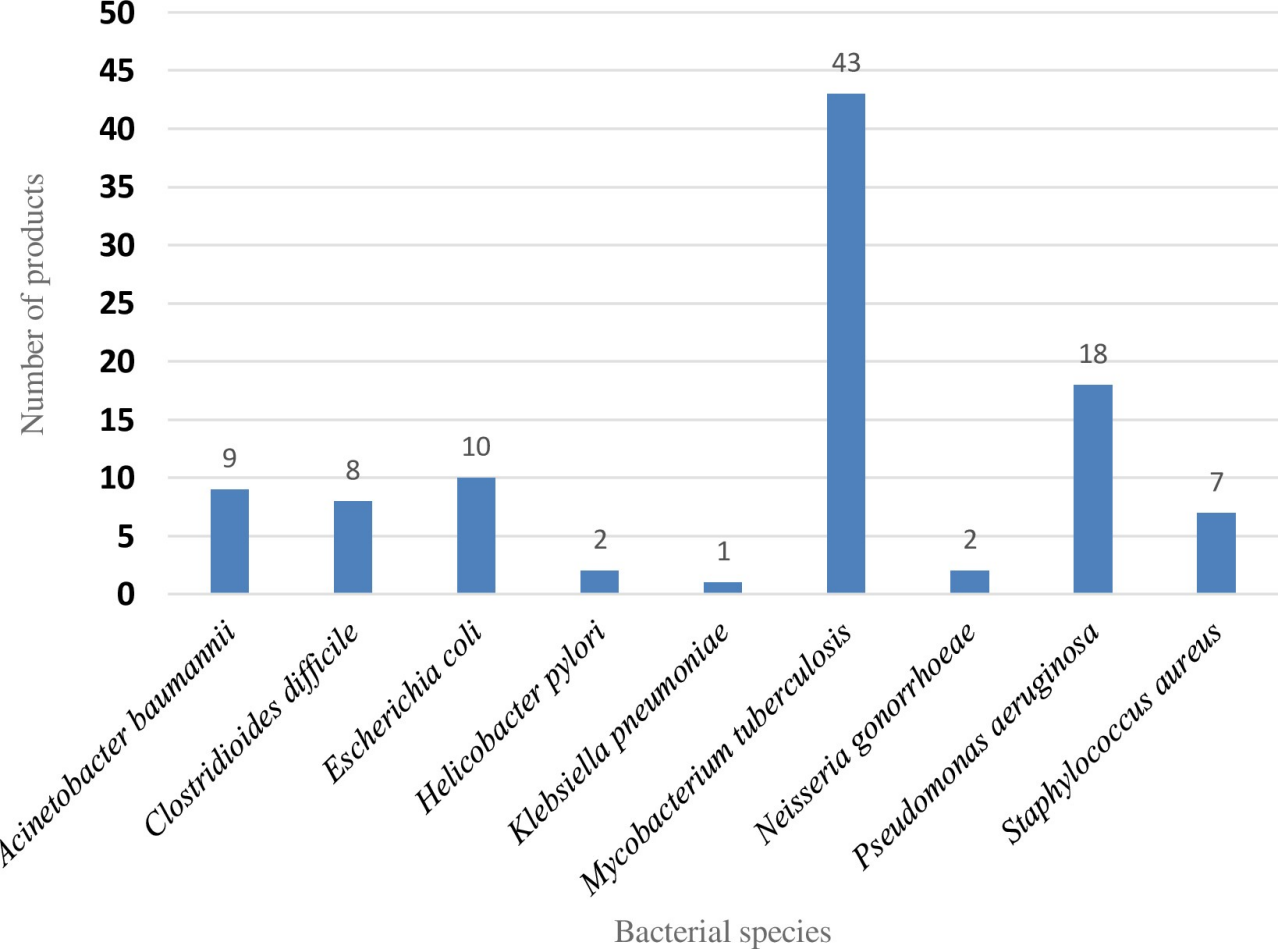
Distribution of pathogens targeted by a single pathogen product (n = 100).

Similarly, early-phase projects often have not refined the specific indication they are targeting. For broader-spectrum agents, this may be because the full understanding of which bacterial species that eventually may be treatable with a safe dose has not been established. Importantly however, a large number of the pre-clinical pipeline projects (n = 100) are focused on a single pathogenic species (Fig 3), which is a distinct shift away from the broad-spectrum antibiotic concept held for many years. Of these species-specific projects, there were) 43 targeting *Mycobacterium tuberculosis*, 18 against *Pseudomonas aeruginosa*, 10 focusing on *Escherichia coli*, 9 directed against *Acinetobacter baumannii* and 8 *Clostridioides difficile* projects.

**Fig 4:**
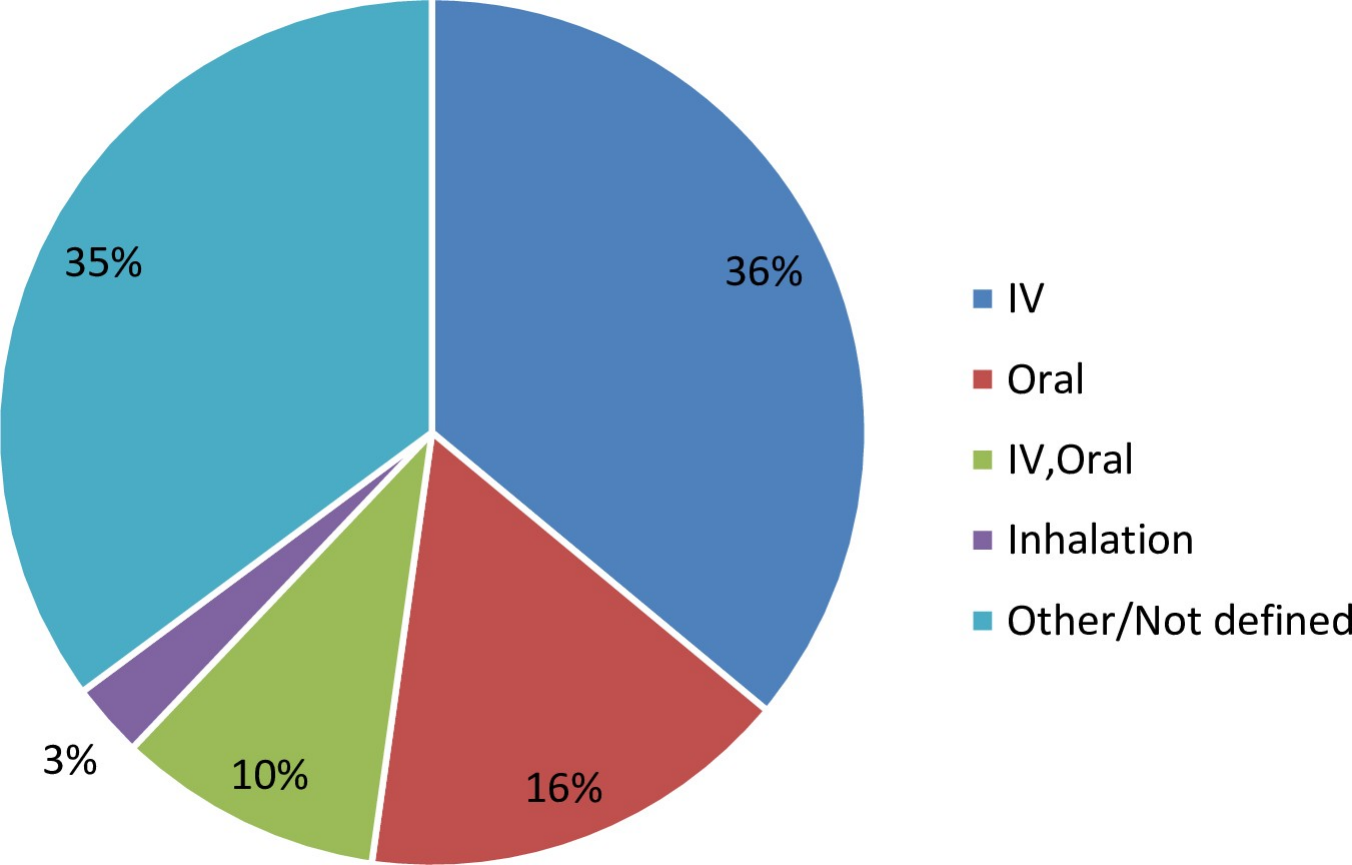
Planned formulations of antibacterial pre-clinical pipeline projects.

The majority of the preclinical pipeline projects (36%) will be formulated for intravenous application, 16% planned for oral application and approximately 10% for both intravenous and oral formulations and 3% inhalation, with the remainder (35%) not disclosing or with an unknown formulation (Fig 4). The fact that approximately one-third of the projects had an unknown administration route is not particularly surprising, as formulation studies may only dictate administration route later in a project. This is particularly true for small molecules where the physiochemical properties of the final compound may be impacted by the medicinal chemistry program that optimizes the other attributes required for project progression.

## Discussion

This is the first review of individual pre-clinical antibacterial pipeline projects and represents that is based on publicly disclosed information. This analysis suggests several strong trends. There is a high degree of innovation in the preclinical pipeline when compared to the clinical antibacterial pipeline [4,8] with respect to the diversity in novel and non-traditional approaches. This may represent a recent shift in thinking among researchers as to how best address the growing threat of antibacterial resistance, particularly in the Gram-negative species. Given that the majority of these approaches are untested from a clinical standpoint, and often cannot follow established regulatory pathways, the failure rate of these non-traditional approaches is expected to be higher than the more traditional direct-acting small molecules. This is notably true for the anti-virulence approaches that in combination with the standard-of-care agent need to demonstrate added clinical benefit under an appropriate new clinical trial design. The data shows an increased focus on products that are targeting Gram-negative pathogens, likely influenced by the WHO priority pathogen list [9]. These two trends are likely responsible for the significant observed shift toward narrower-spectrum products focused on a single pathogen. The most narrow-spectrum agents target *M*. *tuberculosis* which is understandable given both the unique biology of the species and the high unmet medical need of the disease, followed by the critical Gram-negative bacteria identified in the WHO priority pathogens list. This shift could be due to the increase of more species-specific non-traditional approaches, for example phage/phage derived peptides or anti-virulence agents. As these species-specific products progress through the development pipeline they will have to overcome some challenges both in development and clinical use, probably requiring complementary diagnostics for patient stratification during development and for rapidly influencing therapeutic decisions clinically. Finally the current preclinical antibacterial pipeline is relying more heavily on smaller biotechnology companies and academic institutions than ever before to progress new medicines toward clinical use. The market dynamics is currently not favourable for the survival of these SMEs, even if they are successful in bringing a product to the market. The bankruptcy of Achaogen that marketed plazomicin and the recent filing for bankruptcy of Melinta, that had four antibiotics on the market, are examples of the struggle that SMEs can face [10,11]. Greater public engagement is needed to identify new solutions beyond push and pull incentives as well as the need for more major pharmaceutical companies to re-engage in different aspects of antibacterial development.

### Limitations

The review of the pre-clinical antibacterial pipeline is heavily reliant on self-reporting of developer through the WHO DataCol, although steps were taken to clean the data and re-confirm with available online sources. This review is a snapshot in time of the pre-clinical antibacterial pipeline and not an analysis.
